# Effect of Vitamin C Supplementation on C-reactive Protein Levels in Patients Undergoing Hemodialysis: A Randomized, Double Blind, Placebo-Controlled Study

**DOI:** 10.5812/numonthly.13351

**Published:** 2013-11-27

**Authors:** Vajihe Biniaz, Mehdi Sadeghi Shermeh, Abbas Ebadi, Ali Tayebi, Behzad Einollahi

**Affiliations:** 1 Nephrology and Urology Research Center, Baqiyatallah University of Medical Sciences, Tehran, IR Iran; 2 Department of Medical Surgical Nursing, Baqiyatallah University of Medical Sciences, Tehran, IR Iran

**Keywords:** Renal Insufficiency, Chronic, Renal Dialysis, C- Reactive Protein, Ascorbic Acid

## Abstract

**Background:**

Chronic inflammation is the most important cause of cardiovascular disease in patients undergoing hemodialysis, and vitamin C as a major antioxidant which could be effective to suppress inflammation.

**Objectives:**

This study was performed to evaluate the effect of vitamin C supplementation on C-reactive protein levels in patients undergoing hemodialysis.

**Patients and Methods:**

This randomized, placebo-controlled and double-blind trial was conducted on 151 patients on hemodialysis who were divided randomly by lottery method to three identical groups. In the intervention group, 250 mg of vitamin C was injected intravenously immediately at the end of each hemodialysis session three times a week for 8 weeks in a row. In the control group 1, same term of placebo saline was injected, and in the control group 2, no intervention was performed.

**Results:**

A total of 86 (61%) male and 55 female patients with mean hemodialysis duration of 39.74 ± 45.5 months, and a mean age of 61.36 ± 11.46 years-old, participated in this study. Hypertension and diabetes were the most common underlying diseases (79.4%). Median baseline CRP in the intervention, control 1 and control 2 groups were 16.8, 17.8, and 19.4 mg/L respectively. After 2 months, median CRP reduced significantly in the vitamin C group to 10.7 (P = 0.04) vs. 22.6, and 30.6 mg/L in control groups.

**Conclusions:**

Our findings demonstrated that vitamin C supplementation modifies the levels of CRP in patients on hemodialysis.

## 1. Background

End-stage renal disease (ESRD) is one of the most common chronic diseases (1), and a major healthcare problem specifically in developing countries (2). It is reported that the incidence and prevalence of ESRD are on the rise (3). Although patients undergoing hemodialysis, as a maintenance invasive treatment, can live longer (4), they experience diverse complications that noticeably disturb their quality of life (5). According to data, mortality in patients on hemodialysis is almost 9-20 times more than the general population (6), and mainly occurs due to atherosclerotic cardiovascular events (7).

Chronic inflammation, a common disorder in patients on hemodialysis (8) which represented by increased C-reactive protein (9), is the most important cause of cardiovascular disease (10-12), and severely linked with morbidity and mortality in these patients (13, 14). Although inflammation etiology in patients undergoing hemodialysis is varied (15), they put on show the highest level of inflammation likely due to hidden infection associated with arteriovenous fistula or central venous catheters (16), confrontation with endotoxin, and other pollutants and bioincompatible dialysis solution and membrane (17).

C-reactive protein (CRP) is a major biomarker of inflammation and a mediator of atherosclerosis (18). Serum level of CRP increases predominantly during inflammatory processes (19), and is elevated in a large proportion of patients on hemodialysis (20).

There is plenty evidence indicating the predictive value of CRP as a potent and strong independent risk marker for cardiovascular disease (CVD), and its morbidity and mortality (21). Based on these findings, the determination of CRP level and its management as a main marker of inflammatory status of patients undergoing hemodialysis might help to pick out individuals with an high atherothrombotic risk (22), and lead to improved outcomes (23).

Since elevated oxidative stress plays a basic role in inflammation production due to the release of inflammatory mediators (24), and increases free radicals associated with lessening production of essential antioxidants (25) in patients on hemodialysis, vitamin C (ascorbic acid) could be effective as a major antioxidant in inflammation diminution (26).

The antioxidant function of ascorbic acid, which is a water-soluble vitamin in human plasma, has been extensively studied in individuals with and without kidney failure (27). Vitamin C deficiency is a prevalent complication in patients undergoing hemodialysis (28-30), and an important risk factor for increased inflammatory status (24), cardiovascular disease and its mortality, occurs mainly due to loss of the vitamin during dialysis sessions, and dietary restriction of fresh fruits and vegetables to avoid hyperkalemia (31).

## 2. Objectives

Hitherto, different methods have been tested to treat elevated CRP in patients undergoing hemodialysis, but existing data on the effect of vitamin C on serum level of CRP is insufficient and had conflicting results (32-34). This study was performed to evaluate whether vitamin C supplementation could modify CRP levels in patients undergoing hemodialysis.

## 3. Patients and Methods

This randomized, placebo-controlled and double-blind trial was conducted from October 2012 to January 2013 on 151 patients with ESRD undergoing maintenance dialysis in two hemodialysis units in Baqiyatallah and Chamran hospitals of Tehran, Iran. They were randomly distributed by lottery method into three identical groups (simple random sampling); intervention group received vitamin C, control group 1 received placebo, and control group 2 received no intervention. Qualification was not limited to patients with elevated CRP. The sampling frame included all patients with ESRD aged equal or older than 18 years, regular recourse for hemodialysis of three sessions per week, receiving hemodialysis ≥ 6 months, and not taking vitamin C or E at least from 3 months ago. Exclusion criteria included history of renal transplantation for less than one year ago, using anti-inflammatory medications such as immunosuppressive agents, being infected by active infections, getting cancers, smoking and passive smoke exposure, and alcohol consumption.

Of 152 randomized patients, 10 were excluded from the study due to transmission to other dialysis centers, being infected by active infections, getting cancers, death, or their own refusal, and only 141 patients completed the study.

All groups were alike in clinical particulars including age, sex, weight, marital and employment status, length and session of hemodialysis and smoking. The length of dialysis in all of patients was approximately 4 hours, and its occasions were 3 times a week with resemble KT/V (it a scale used to assess the adequacy of hemodialysis and peritoneal dialysis treatment). The KT/V did not change during the research.

Prior ethical approval was obtained from the institutional ethical committee at Baqiyatallah University of Medical Sciences, Tehran, Iran. A justification letter was sent to two hemodialysis units to gain permission to collect the data granted by these units. Oral and written consents were obtained from all those who participated in the study. Written consents were obtained after informing each participant about the study purposes, the “confidentiality” of their information, and the possibility to refuse the test procedure at any stage of it.

A demographic questionnaire was developed by the researcher to record age, gender, marital and education status, number of children, employment status, income, weight, smoking history, nephropathy cause, and length of time receiving dialysis. Serum levels of C-reactive protein were measured at the beginning, and at the end of the intervention.

In intervention group, 250 mg of vitamin C was injected intravenously immediately at the end of each hemodialysis session three times a week for 8 weeks in a row. In the control group 1, same term of placebo saline was injected, and in control group 2 no intervention was performed. Although the recommended amount for vitamin C intake in patients on hemodialysis is 100-200 mg per day (35), only 250 mg of vitamin C three times a week, lower than the safe dosage recommended by NIH (28) was prescribed to prevent oxalosis.

All data were shown as mean ± SD. The X^2^-test, T-test and ANOVA were used in this study. The data was analyzed by SPSS software (version 18). The researcher decided to receive only a 5% error in rejecting the full hypothesis at the 95% confidence interval. The significance level was put at 0.05.

## 4. Results

Complete data was gathered for 141 patients on hemodialysis (86 male, 55 female) with a mean age of 61.36 ± 11.46 years, and mean hemodialysis duration of 39.74 ± 45.5 months. There was no significant association between age and duration on hemodialysis with CRP levels by using independent sample t-test.

The most common etiology of nephropathy was hypertension (38.3% hypertension, and 26.2% hypertension and DM simultaneously). The highest mean CRP level was seen in individuals with hypertension, but there was no significant correlation between nephropathy causes and CRP levels.

The mean duration of dialysis sessions was 3.8 h. All groups, with and without vitamin C supplementation, were similar in sex proportion, age, main clinical characteristics, and mean time on hemodialysis. Educational level was primary-secondary in 51.8% of participants. There was no significant correlation between educational level and serum CRP levels. Additional demographic characteristics of different groups’ patients according to variable kind are shown in Tables 1 and 2. 

CRP > 6 and CRP > 10 mg/L were observed in 41.1% and 34.8% of the patients, respectively. Median baseline CRP in the case, control 1 and control 2 groups were 16.8, 17.8, and 19.4 mg/L respectively. As seen in Table 3, patients of the three groups did not have any significant difference regarding serum level of CRP at baseline. After 2 months, median CRP reduced significantly in the vitamin C group to 10.7 (P = 0.04) vs. 22.6 and 30.6 mg/L in control groups. 

**Table 1.** Baseline Qualitative Characteristics of the Respondents

Variables	Group			X^2^-test
	Vitamin C, No. (%)	Placebo, No. (%)	Control, No. (%)	
**Gender**				0.83
Male	32 (22.7)	34 (24.1)	20 (14.2)	
Female	23 (16.3)	21 (14.9)	11 (7.8)	
**Education**				0.88
Primary-secondary	29 (20.5)	30 (21.3)	14 (9.9)	
College/university-level	26(18.4)	25 (17.7)	17 (12.1)	
**Marital status**				0.3
Married	49 (34.8)	43 (30.5)	28 (19.9)	
Single/widow	6 (4.3)	12 (8.5)	3 (2.1)	
**Employment**				0.95
Employed	4 (2.8)	3 (2.1)	3 (2.1)	
Retired	28 (19.9)	32 (22.7)	17 (12.1)	
Jobless/housekeeper	23 (16.3)	20 (14.2)	11 (7.8)	
**Smoking**				0.45
Yes	1 (0.7)	0	0	
No	54 (38.3)	55 (39)	31 (22)	
**Nephropathy cause**				0.2
HTN^a^	19 (13.5)	20 (14.2)	12 (8.5)	
DM^a^	5 (3.5)	7 (5)	8 (5.7)	
HTN and DM	17 (12.1)	16 (11.3)	8 (5.7)	
Others	14 (9.9)	12 (8.5)	3 (2.1)	

^a^ Abbreviations: DM, diabetes mellitus; HTN, hypertention.

**Table 2.** Baseline Quantitative Characteristics of the Respondents

Variables	Group			ANOVA
	Vitamin C, Mean ± SD	Placebo, Mean ± SD	Control, Mean ± SD	
**Age, y**	59.4 ± 11.8	62.75 ± 10.85	62.39 ± 11.77	0.26
**Dialysis vintage, mo**	47.69 ± 45.7	32.83 ± 26.51	34.45 ± 34.47	0.08
**Body weight, Kg**	68.2 ± 11.87	71.8 ± 13.33	67.8 ± 10.69	0.21

**Table 3.** Variables Alterations in Different Stages of Study

Variable	Time	Group			ANOVA
		Vitamin C, Mean ± SD	Placebo, Mean ± SD	Control, Mean ± SD	
**CRP levels**					
	1	16.84 ± 27.9	17.79 ± 27.6	19.4 ± 26.7	0.92
	2	10.78 ± 25.4	22.66 ± 38.5	30.66 ± 46.4	0.04

## 5. Discussion

Our findings demonstrated that vitamin C supplementation modifies the levels of CRP in patients undergoing hemodialysis. Results of the present study were in accordance and supported by earlier studies such as Block (36), and Gholipour (37), but these findings were contrary with Fumeron study (38). He suggested that oral vitamin C cannot modify the levels of CRP in patients undergoing hemodialysis. One possible explanation for the lack of effectiveness of supplementation with oral vitamin C in modifying the levels of CRP in patients undergoing hemodialysis could be related to the route of vitamin C prescription (oral vs. intravenous), so that could foresee higher plasma vitamin C concentrations in patients on hemodialysis after intravenous prescription. According to data intravenous prescription increases serum level of vitamin C more than similar doses of oral vitamin C (39), and this increased serum level is extremely associated with lessen oxidative stress and CRP levels (40). Also, small sample size (33 people) and no usage of placebo, randomization, and control group in Fumeron study could be other reasons for it. Consistent with earlier literature, our study indicated that hypertension and diabetes were the most common causes of nephropathy (hypertension and diabetes 79.4%).

Unlike findings from some studies, in which more than 50% of patients on hemodialysis were jobless, our data suggested that 61.7% of participants were retired or employed, and only 4.3% were unemployed. This could indicate that the government and insurance companies have an appropriate support of patients on hemodialysis in Iran.

Although results of Helal study showed that there was a significant correlation between age above 40 years and more than 5 years duration on hemodialysis with serum hs-CRP (41), in this study an independent samples t-test confirmed that there was no significant association between CRP level with age and duration on hemodialysis.

According to data, the incidence of CKD is higher in men and people older than 45 years. In the present study, while the mean age of patients was 61.36 (SD 11.46) years, most of them (61%) were male, and 91.5% were older than 45 years. 

Patients on hemodialysis are at risk for low levels of serum vitamin C (42, 43). Vitamin C is a water-soluble vitamin which can be reduced by regular hemodialysis (44). Dietary restrictions following fear related to hyperkalemia (45), concern oxalosis, wasting several hundred mg of vitamin C during one session of hemodialysis (46), and fast catabolism lead to vitamin C deficiency in patients on hemodialysis. Although normal range of serum vitamin C is 30-60 μM, most patients undergoing hemodialysis have serum levels of vitamin C less than 10 μM, and even some of them have less than 2 μM (47). Moreover, chronic inflammation due to the release of inflammatory mediators in patients on hemodialysis leads to reduced production of essential antioxidants and increased oxidative stress (26), and it increases free radicals associated with vitamin C deficiency as an important antioxidant (25). Therefore vitamin C deficiency may play an important role in increased inflammatory status in these patients.

Even if 60 to 100 mg of vitamin C in an individual with normal kidney function is sufficient for health maintenance, it may not be adequate in a patient on hemodialysis due to the mentioned reasons. The recommended amounts for vitamin C in patients undergoing hemodialysis are 100-200 mg per day, which almost none of patients on hemodialysis consume (48).

Albeit existing data about the effect of vitamin C on CRP levels in patients on hemodialysis were contradictory, our data confirmed that there was a reverse association between serum levels of vitamin C and CRP (49, 50). CRP levels decreased more obviously in intervention group who received supplemental vitamin C than the control group (P = 0.028). But no significant difference in CRP levels was observed between the placebo and witness groups.

Not measuring plasma levels of vitamin C before, and after the study, and not specifying the patients who had vitamin C deficiency before the study were the limitations in our study which burden the ability to generalize the findings. Removing these limitations was not a feasible option due to the financial costs, and the limitations of laboratories capable of providing the circumstance for this test. It is recommended to perform studies with longer-term use of vitamin C, and larger sample sizes.

Conclusions: Our findings demonstrated that there was a reverse relation between supplemental vitamin C and CRP levels, and vitamin C supplementation could modify the levels of CRP in patients on hemodialysis. Serial determinations of CRP are more effective for a better prediction of the inflammatory state of patients on chronic hemodialysis than scattered single time-point measurements. Vitamin C supplementation with its CRP-lowering effect could be a simple and useful method in modifying inflammation status, and a potential goal for reducing cardiovascular disease risk in patients on hemodialysis. Therefore, further investigations with longer-term use of vitamin C and larger sample sizes are recommended.
